# Risk factors for sporadic listeriosis in Beijing, China: a matched case–control study

**DOI:** 10.1017/S0950268821002673

**Published:** 2022-02-21

**Authors:** Yan-Lin Niu, Tong-Yu Wang, Xiao-Ai Zhang, Yun-Chang Guo, Ye-Wu Zhang, Chao Wang, Yang-Bo Wu, Jin-Ru Jiang, Xiao-Chen Ma

**Affiliations:** 1 Institute for Nutrition and Food Hygiene, Beijing Center for Disease Prevention and Control, Beijing 100013, China; 2 Research Center for Preventive Medicine of Beijing, Beijing 100013, China; 3 Division of Foodborne Disease Surveillance, China National Center for Food Safety Risk Assessment, Beijing 100013, China; 4 Center for Public Health Surveillance and Information Service, Chinese Center for Disease Control and Prevention, Beijing 100013, China

**Keywords:** Case–control study, listeriosis, risk factor

## Abstract

Listeriosis is a rare but serious foodborne disease caused by *Listeria monocytogenes*. This matched case–control study (1:1 ratio) aimed to identify the risk factors associated with food consumption and food-handling habits for the occurrence of sporadic listeriosis in Beijing, China. Cases were defined as patients from whom *Listeria* was isolated, in addition to the presence of symptoms, including fever, bacteraemia, sepsis and other clinical manifestations corresponding to listeriosis, which were reported via the Beijing Foodborne Disease Surveillance System. Basic patient information and possible risk factors associated with food consumption and food-handling habits were collected through face-to-face interviews. One hundred and six cases were enrolled from 1 January 2018 to 31 December 2020, including 52 perinatal cases and 54 non-perinatal cases. In the non-perinatal group, the consumption of Chinese cold dishes increased the risk of infection by 3.43-fold (95% confidence interval 1.27–9.25, *χ*^2^ = 5.92, *P* = 0.02). In the perinatal group, the risk of infection reduced by 95.2% when raw and cooked foods were well-separated (*χ*^2^ = 5.11, *P* = 0.02). These findings provide important scientific evidence for preventing infection by *L. monocytogenes* and improving the dissemination of advice regarding food safety for vulnerable populations.

## Introduction

Listeriosis, caused by the Gram-positive facultative intracellular bacterium *Listeria monocytogenes*, is a rare but serious disease with a high mortality rate of 20–40% [1, 2]. Most patients with listeriosis are infected through food contamination, as well via transplacental mother-to-infant transmission or infection during childbirth [3]. The populations vulnerable to *L. monocytogenes* include the elderly, pregnant women and their unborn foetuses, newborn babies and immunocompromised persons [4]. The clinical manifestations of listeriosis vary, including bacteraemia, sepsis and focal infections in organ systems and prostheses [4]. Infection in pregnant women can result in adverse events, including abortion, stillbirth, premature delivery or neonatal meningitis [5].

The estimated incidence of listeriosis is three to six patients per 1 million population per year globally [6]. In China, the overall mortality rate of listeriosis is approximately 26%, and it is 24% in non-perinatal patients, with 33–46% of pregnancy-related listeria infections ending in abortions and/or neonatal deaths [7, 8]. Previous studies have investigated the risk factors associated with food consumption and food-handling habits; these studies reported that the high-risk food included ready-to-eat foods, delicatessen-style processed foods and fruit and vegetable products, and that the high-risk behaviours for listeriosis pregnant women included a history of contact with domestic animals [5, 9]. However, it is difficult to identify food associated with listeriosis due to the ubiquitous and cold-tolerant nature of *L. monocytogenes*, its long and varying incubation period and the restriction of severe illness to mainly the vulnerable populations [10, 11]. Most high-risk foods were identified in outbreaks, while the results of case–control studies investigating the risk factors in sporadic patients have been inconsistent [4].

The type of food consumed and the food-handling habits associated with listeriosis in Chinese individuals are unknown as no outbreaks have been identified in China to date [7]. In addition, food consumption patterns and food-handling habits in China differ significantly from those in Western countries. Therefore, the findings of studies conducted in other countries may not be appropriate references for the Chinese population. Thus, this matched case–control study aimed to identify the risk factors for listeriosis associated with food consumption and food-handling habits in Beijing, China.

## Methods

Surveillance for human listeriosis in Beijing, China, was launched in 2013. According to the monitoring scheme issued by Beijing Municipal Health Commission, medical institutions with the capacity of diagnosis and treatment of foodborne diseases were required to report cases of listeriosis via the Beijing Foodborne Disease Surveillance System when biospecimens of patients with clinical symptoms were positive for *L. monocytogenes*. All the *L. monocytogenes* isolates were first identified using the VITEK 2-compact system (bioMérieux, Lyons, France) or matrix-assisted laser desorption/ionisation time of flight mass spectrometry (Bruker, Leipzig, Germany) and were further checked by polymerase chain reaction targeting *hly* fragments specific to *L. monocytogenes* [12]. The present matched case–control study (1:1 ratio) enrolled all cases compatible with the following criteria between 1 January 2018 and 31 December 2020: cases were defined as patients from whom *Listeria* was isolated, in addition to the presence of symptoms such as fever, bacteraemia, sepsis and other clinical manifestations corresponding to listeriosis. Non-perinatal cases were defined as those who were not pregnant women or newborns. Perinatal cases were defined as patients who were pregnant women or newborns, as well as cases of foetal loss. If both the mother and her newborn or foetus were positive for *L. monocytogenes*, they were counted as one case. Cases were excluded if they were (1) asymptomatic, except for perinatal mothers; (2) part of an outbreak associated with an identified food vehicle that was identified by whole genome sequencing and epidemiological investigation; (3) unable to be contacted within 4 weeks of the culture date and (4) when the treating doctor or family member refused participation. Controls were selected from the same hospital as matched cases. The general matched conditions included sex and age within 5 years. In addition, for non-perinatal cases, the controls were required to have similar underlying immune conditions as that of the matched cases. Specifically, hypoimmunity in the case–control pairs was required to have similar causes, such as organ transplantation, tumour, renal disease, etc., to reduce the influence of the possible changes in eating habits caused by a particular disease. For perinatal cases, the gestational weeks of the controls were within 2 weeks of all cases. All controls were recruited within 4 weeks of conducting the case interviews. Cases were excluded if matched controls could not be found. Besides the symptomatic treatment, standard antimicrobial treatment for listeriosis, typically including IV ampicillin and gentamicin, would be indicated to the cases by the doctors.

Face-to-face interviews with cases or their surrogates and controls were conducted by staff from the Centers for Disease Control and Prevention using a standard structured questionnaire. Basic patient information and the possible risk factors associated with food consumption and food-handling habits were obtained from the patient or a surrogate. Combined with dietary habits and Chinese characteristics, consumption of 11 food items, identified as having a high risk of *Listeria* contamination in previous studies, were investigated in the 4 weeks before the specimen collection date for cases, in the corresponding 4 weeks for perinatal controls and in the 4 weeks before the interview date for non-perinatal controls. Among these food items, Chinese cold dishes are a traditional food in China and can be mainly classified as bean, vegetable or meat products. Dishes such as Chinese cucumber salad are popular because they are simple to prepare, tasty and nutritious. Cold dishes may be prepared from cooked and/or raw ingredients in advance, are stored at room temperature before serving, and are not generally reheated before consumption [13, 14]. Cold dishes can be purchased from supermarkets or prepared at home. However, their inherent properties and preparation processes make them susceptible to contamination by a variety of pathogens or spoilage bacteria. Compared to Chinese cold dishes, Western-style salads are similar cold dishes prepared using Western processing methods. In addition, this study investigated eight types of food-handling habits – whether raw and cooked foods were well separated, the frequency of refrigerator cleaning, etc. The ethics committee of the China National Center for Food Safety Risk Assessment approved the study. All the cases and controls provided informed consent before enrolment.

Descriptive analyses were conducted for both perinatal and non-perinatal cases as well as controls. Univariable analysis was performed for all food consumption and living habit variables. Univariable odds ratios (ORs) and 95% confidence intervals (CIs) were computed using logistic regression adjusted for the matching factors. Multivariable logistic regression modelling was used to investigate the independent relationships between risk factors and listeriosis while adjusting for the potential confounding effects of other factors. A forward stepwise selection strategy was employed and all variables in the univariable analysis were included in the multivariable logistic analysis. Due to the limited sample size in this study, food consumption and food-handling habits were analysed in separate models instead of a combined model to obtain robust estimates. In this study, *P* < 0.05 was considered statistically significant. All reported *P*-values were two-tailed. All analyses were performed using IBM SPSS Statistics for Windows, version 23.0 (IBM Corp., New York, USA).

## Results

### Characteristics of the cases and controls

One hundred and thirty-four cases were reported from 1 January 2018 to 31 December 2020, including 63 perinatal cases and 71 non-perinatal cases. Eight cases could not be contacted within 4 weeks of the culture date and three cases refused participation in the investigation; therefore, these 11 cases were excluded from the study. An additional 17 cases were excluded due to the absence of matched controls. No outbreak was identified during the study period. Finally, 106 cases were enrolled during the study period, corresponding to 106 pairs of cases and controls (Table 1). The total response rates for the cases and controls were both 79.10% (106/134). However, the response rates for the cases and controls were only 24.00% (6/25) and 28.00% (7/25) in 2020, mainly due to the coronavirus disease 2019 epidemic. This study enrolled a total of 52 perinatal cases and 54 non-perinatal cases. Among the perinatal cases, 40 were identified by cultures of samples from pregnant and birth-giving women, while 12 cases were from newborns. Based on the age of the mother, the age range in the perinatal group was 23 ± 13 years, compared to 50 ± 25 years in the non-perinatal group. The non-perinatal group comprised of 26 men and 28 women.

**Table 1.** Basic information on listeriosis cases and controls from 2018 to 2020 in Beijing, China

Year			2018	2019	2020	Total
Reported cases	Perinatal	Pregnant and birth-giving women	18	23	10	51
		Newborns	3	7	2	12
	Non-perinatal		29	29	13	71
	Total		50	59	25	134
Enrolled cases	Perinatal	Pregnant and birth-giving women	18	21	1	40
		Newborns	3	8	1	12
	Non-perinatal		25	25	4	54
	Total		46	54	6	106
Excluded cases	Lose track		4	1	3	8
	Refused participation		0	3	0	3
	Total		4	4	3	11
Enrolled controls	Perinatal		20	29	3	52
	Non-perinatal		23	27	4	54
	Total		43	56	7	106
No matched controls			0	1	16	17
Response rate for cases	Perinatal		100.00%	96.67%	16.67%	82.54%
	Non-perinatal		86.21%	86.21%	30.77%	76.06%
	Total		92.00%	91.53%	24.00%	79.10%
Response rate for controls	Perinatal		95.24%	96.67%	25.00%	82.54%
	Non-perinatal		79.31%	93.10%	30.77%	76.06%
	Total		86.00%	94.92%	28.00%	79.10%

### Univariate analysis

The results of the univariate analysis showed no significant differences in any high-risk foods in the perinatal group. In the non-perinatal group, Chinese cold dishes were identified as a high-risk food for *L. monocytogenes* contamination, with an OR value of 2.75 (*χ*^2^ = 6.00, *P* = 0.01) (Table 2).

**Table 2.** Food-related risk factors for sporadic listeriosis in Beijing, China

Consumption	Perinatal				Non-perinatal			
	Cases	Controls	OR (95% CI)		Cases	Controls	OR (95% CI)	
			Univariate	Multivariate			Univariate	Multivariate
Raw vegetables	34 (65.38%)	38 (73.08%)	0.84 (0.47–1.49)	0.84 (0.46–1.54)	34 (62.96%)	33 (61.11%)	1.11 (0.45–2.73)	0.93 (0.26–3.27)
Cooked meat products	34 (65.38%)	34 (65.38%)	1.00 (0.57–1.77)	0.97 (0.54–1.76)	34 (62.96%)	33 (61.11%)	1.10 (0.47–2.59)	0.60 (0.18–2.07)
Raw aquatic products	3 (5.77%)	6 (11.54%)	0.65 (0.20–2.07)	0.56 (0.17–1.86)	2 (3.70%)	8 (14.81%)	0.14 (0.02–1.16)	0.16 (0.01–2.43)
Sushi	7 (13.46%)	7 (13.46%)	1.00 (0.45–2.22)	0.42 (0.13–1.33)	3 (5.56%)	6 (11.11%)	0.25 (0.03–2.24)	0.36 (0.01–10.26)
Fruit	48 (92.31%)	51 (98.08%)	0.61 (0.22–1.68)	0.89 (0.46–1.72)	47 (87.04%)	51 (94.44%)	0.43 (0.11–1.66)	0.38 (0.08–1.84)
Freshly prepared drinks	13 (25.00%)	14 (26.92%)	0.95 (0.51–1.78)	1.12 (0.62–2.03)	7 (12.96%)	5 (9.26%)	1.50 (0.42–5.32)	1.47 (0.27–7.84)
Ice cream	21 (40.38%)	17 (32.69%)	1.18 (0.68–2.05)	1.44 (0.71–2.90)	10 (18.52%)	7 (12.96%)	1.50 (0.53–4.21)	3.21 (0.63–16.35)
Chinese cold dishes	36 (69.23%)	34 (65.38%)	1.09 (0.61–1.97)	1.40 (0.68–2.87)	31 (57.41%)	17 (31.48%)	2.75 (1.22–6.18)	3.43 (1.27–9.25)
Western-style salad	10 (19.23%)	6 (11.54%)	1.31 (0.66–2.61)	1.61 (0.74–3.51)	3 (5.56%)	4 (7.41%)	0.67 (0.11–3.99)	1.76 (0.13–24.40)
Raw milk	2 (3.85%)	0 (0.00%)	2.04 (0.50–8.39)	0.84 (0.46–1.54)	5 (9.26%)	2 (3.70%)	4.00 (0.45–35.79)	2.94 (0.23–37.55)
Cheese	10 (19.23%)	5 (9.62%)	1.41 (0.71–2.82)	0.97 (0.54–1.76)	7 (12.96%)	5 (9.26%)	1.40 (0.44–4.41)	1.03 (0.18–5.79)

OR, odds ratio; CI, confidence interval.

Regarding food-handling habits, separation of raw and cooked food and the frequency of refrigerator cleaning were factors that influenced the risk of *L. monocytogenes* infection in the perinatal group (Table 3). Both of these were protective factors against listeriosis, with ORs of 0.15 (*χ*^2^ = 9.39, *P* < 0.01) and 0.05–0.21, respectively, indicating that the higher the frequency of cleaning, the less likely the food was to be infected. Similarly, the results in the non-perinatal group showed the protective effects of separating raw and cooked foods, with an OR of 0.33 (*χ*^2^ = 5.43, *P* = 0.02). In addition, handling the cutting board properly after cutting raw meat, avoiding the handling of live poultry and keeping pets were also protective factors against *L. monocytogenes* infection.

**Table 3.** Risk factors associated with living habits for sporadic listeriosis in Beijing, China

Dietary habits	Perinatal				Non-perinatal			
	Cases	Controls	OR (95% CI)		Cases	Controls	OR (95% CI)	
			Univariate	Multivariate			Univariate	Multivariate
Handling of poultry meat								
No^a^	17 (32.69%)	22 (42.31%)	–	–	18 (33.33%)	26 (48.15%)	–	–
Yes	35 (67.31%)	30 (57.69%)	1.71 (0.68–4.35)	2.33 (0.48–11.40)	36 (66.67%)	28 (51.85%)	2.00 (0.86–4.67)	1.35 (0.59–3.10)
Hand washing after handling poultry meat								
Na	0 (0.00%)	0 (0.00%)	–	–	18 (33.33%)	26 (48.15%)	–	–
Almost always	26 (74.29%)	25 (83.33%)	–	–	26 (48.15%)	16 (29.63%)	–	–
Often	9 (25.71%)	5 (16.67%)	–	–	9 (16.67%)	9 (16.67%)	–	–
Occasionally	0 (0.00%)	0 (0.00%)	–	–	0 (0.00%)	2 (3.70%)	–	–
Never	0 (0.00%)	0 (0.00%)	–	–	1 (1.85%)	1 (1.85%)	–	–
Washing cutting boards after handling poultry meat								
Almost always	22 (62.86%)	24 (80.00%)	–	–	29 (80.56%)	16 (57.14%)	–	–
Often	6 (17.14%)	3 (10.00%)	–	–	6 (16.67%)	8 (28.57%)	–	–
Occasionally	5 (14.29%)	1 (3.33%)	–	–	1 (2.78%)	3 (10.71%)	–	–
Never	2 (5.71%)	2 (6.67%)	–	–	0 (0.00%)	1 (3.57%)	–	–
Separation of raw and cooked foods								
No^a^	30 (57.69%)	13 (25.00%)	–	–	24 (44.44%)	12 (22.22%)	–	–
Yes	22 (42.31%)	39 (75.00%)	0.15 (0.05–0.51)	0.05 (0.004–0.67)	30 (55.56%)	42 (77.78%)	0.33 (0.13–0.84)	0.79 (0.37–1.66)
Handling of the cutting board after cutting raw meat								
Use without any processing	2 (3.85%)	5 (9.62%)	0.80 (0.06–9.88)	0.59 (0.01–62.32)	0 (0.00%)	0 (0.00%)	–^#^	–^#^
Only wipe with a rag before use	1 (1.92%)	2 (3.85%)	0.80 (0.06–9.88)	0.32 (0.001–84.85)	0 (0.00%)	3 (5.66%)	–^#^	–^#^
Rinse with running water only before use	23 (44.23%)	19 (36.54%)	2.07 (0.74–5.81)	0.93 (0.01–94.85)	24 (45.28%)	12 (22.64%)	7.84 (1.66–37.01)	1.16 (0.51–2.61)
Use the other side	4 (7.69%)	0 (0.00%)	–^#^	–^#^	1 (1.89%)	1 (1.89%)	2.80 (0.11–71.62)	0.92 (0.09–9.07)
Switch to another cutting board for cooked food^a^	17 (32.69%)	26 (50.00%)	–	–	23 (43.40%)	35 (66.04%)	–	–
Continue to use after cleaning with detergent	5 (9.62%)	0 (0.00%)	–^#^	–^#^	5 (9.43%)	2 (3.77%)	10.25 (1.18–89.27)	1.67 (0.42–6.59)
Frequency of refrigerator cleaning								
<1 time/year^a^	21 (40.38%)	9 (17.31%)	–	–	18 (33.33%)	17 (31.48%)	–	–
<1–2 times/year	24 (46.15%)	19 (36.54%)	0.40 (0.12–1.35)	0.23 (0.04–1.46)	22 (40.74%)	22 (40.74%)	0.95 (0.39–2.33)	1.26 (0.60–2.63)
<3–5 times/year	5 (9.62%)	12 (23.08%)	0.21 (0.05–0.87)	0.52 (0.08–3.25)	8 (14.81%)	9 (16.67%)	0.82 (0.23–2.86)	0.88 (0.22–3.53)
>5 times/year	2 (3.85%)	12 (23.08%)	0.05 (0.01–0.45)	0.20 (0.01–2.98)	6 (11.11%)	6 (11.11%)	0.97 (0.21–4.50)	0.88 (0.24–3.25)
Consumption of leftovers								
No^a^	25 (48.08%)	30 (57.69%)			21 (38.89%)	21 (38.89%)		
Yes	27 (51.92%)	22 (42.31%)	1.71 (0.68–4.35)	1.66 (0.28–10.00)	33 (61.11%)	33 (61.11%)	1.00 (0.38–2.66)	0.45 (0.18–1.12)
Heating leftovers before consumption								
Na	15 (28.85%)	30 (57.69%)	–	–	0 (0.00%)	0 (0.00%)	–	–
Almost always	22 (42.31%)	15 (28.85%)	–	–	24 (57.14%)	24 (72.73%)	–	–
Often	5 (9.62%)	2 (3.85%)	–	–	14 (33.33%)	4 (12.12%)	–	–
Occasionally	5 (9.62%)	5 (9.62%)	–	–	1 (2.38%)	5 (15.15%)	–	–
Never	5 (9.62%)	0 (0.00%)	–	–	3 (7.14%)	0 (0.00%)	–	–
Banquet attendance								
No^a^	42 (80.77%)	44 (84.62%)	–	–	46 (85.19%)	49 (90.74%)	–	–
Yes	10 (19.23%)	8 (15.38%)	1.29 (0.48–3.45)	1.15 (0.28–4.67)	8 (14.81%)	5 (9.26%)	1.60 (0.52–4.89)	0.64 (0.24–1.72)
History of contact with live poultry								
No^a^	50 (96.15%)	50 (96.15%)	–	–	45 (83.33%)	52 (96.30%)	–	–
Yes	2 (3.85%)	2 (3.85%)	1.00 (0.14–7.10)	0.35 (0.02–7.03)	9 (16.67%)	2 (3.70%)	4.50 (0.97–20.83)	1.65 (0.58–4.74)
Pet ownership								
No^a^	46 (88.46%)	47 (90.38%)	–	–	36 (66.67%)	48 (88.89%)	–	–
Yes	6 (11.54%)	5 (9.62%)	1.20 (0.37–3.93)	0.58 (0.07–4.97)	18 (33.33%)	6 (11.11%)	4.00 (1.34–11.96)	1.31 (0.59–2.92)

OR, odds ratio; CI, confidence interval.

a Reference group in the model.

# Credible estimates can not be obtained due to the limited sample size.

### Multivariable analysis

The results of multivariable analysis showed that no high-risk foods were associated with listeriosis in the perinatal group, while Chinese cold dishes were a high-risk food for infection by *L. monocytogenes* in the non-perinatal group, which increased the risk of infection by 3.43-fold (95% CI 1.27–9.25, *χ*^2^ = 5.92, *P* = 0.02) (Table 2).

Regarding food-handling habits, multivariable analysis showed that the separation of raw and cooked foods was the key influencing factor associated with listeriosis in the perinatal group. When the raw and cooked foods were well-separated, the risk of infection reduced by 95.2% (*χ*^2^ = 5.11, *P* = 0.02). However, no dietary habit was found to influence the risk of listeriosis in the non-perinatal group (Table 3).

## Discussion

This is the first case–control study of sporadic listeriosis in Beijing, China. This study aimed to identify the risk factors for listeriosis associated with food consumption and food-handling habits, provide important scientific evidence for preventing *L. monocytogenes* infection and improve the dissemination of advice regarding food safety for vulnerable populations. The results showed that Chinese cold dishes and the separation of raw and cooked food may be possible risk factors for *L. monocytogenes* infection in non-perinatal and perinatal patients, respectively.

The results of this study identified Chinese cold dishes as a high-risk food for listeriosis. Other studies on the prevalence of *L. monocytogenes* in food products in China reported that, on average, *L. monocytogenes* was found in 5.54% of the sampled products and 3.65% of the sampled Chinese cold dishes [15], thus supporting our conclusion regarding food contamination by *L. monocytogenes*. Chinese cold dishes are a commonly consumed food in China, especially in the summer, which is a high-risk season for listeriosis. Most Chinese cold dishes are manufactured from raw vegetables and cooked meat and are not heated during manufacturing or before consumption, making them susceptible to contamination by foodborne pathogens, including *L. monocytogenes*. However, we did not identify any other high-risk foods in either non-perinatal or perinatal cases of listeriosis. Due to the limited number of related studies conducted in China, comparisons with similar designs could not be performed. Similar studies on possible high-risk foods in the USA, UK, Australia and Germany reported statistical associations with different Western foods, including hummus and ice milk [16], ready-to-eat beef, smoked fish, prawns, milk, butter, cheese and mixed salads [4, 5, 17]; however, these foods are seldom consumed in China owing to traditional Chinese dietary habits. Moreover, it is challenging to identify high-risk foods using a case–control study design that spans several years compared to that in a single outbreak, as the prevalence of *L. monocytogenes* in various types of food may vary over time [5].

The results of this study identified the separation of raw and cooked food as a risk factor for food-handling habits in the perinatal group. The prevalence of *L. monocytogenes* in raw meat and poultry can exceed 11%, which is much higher than that in cooked meat [15]. Not separating the raw and cooked food during processing and storage may result in contamination by *L. monocytogenes*, which increases the risk of infection in humans. No other food-handling habits were associated with *L. monocytogenes* infection in this study. One possible reason may be recall bias during the interview. In addition, we found that several persons were often responsible for patients' daily diets. Inaccurate information obtained from the interviewers and changing behaviours of the different respondents may also explain our failure to identify other high-risk food-handling habits.

Despite the strengths, this study has some limitations. First, it was difficult for interviewees to recall their food consumption more than 4 weeks prior, which may make them more likely to report their usual food preferences rather than their exact exposures. Second, the logistic analysis did not consider socio-economic status, despite its importance as a confounding factor for the risk of *L. monocytogenes* infection, which may have affected the study results. In addition, multiple comparisons were made among the risk factors with no adjustment of the alpha value, which may have increased the risk of type I errors.

Due to the ubiquity of microorganisms in the environment and the psychrotrophic nature of *L. monocytogenes*, this microorganism can contaminate a variety of foods, with improper food-handling habits further increasing the risk for infection [18]. Evidence-based health advice on food consumption and food-handling habits for populations vulnerable to *L. monocytogenes* infection is urgently needed. Although *L. monocytogenes* infection cannot be prevented entirely, public health education regarding food safety may be very helpful. Particularly, for pregnant women and immunocompromised individuals, an educational programme on avoiding *L. monocytogenes* infection by raising awareness of high-risk foods, such as Chinese cold dishes, and maintaining good health habits is necessary to increase their understanding of the importance of personal protection.

## Data Availability

The data are not publicly available due to their containing information that could compromise the privacy of research participants.
